# *Metarhizium robertsii* protease and conidia production, response to heat stress and virulence against *Aedes aegypti* larvae

**DOI:** 10.1186/s13568-021-01326-1

**Published:** 2021-12-13

**Authors:** Juliana M. Ferreira, Salorrane M. N. Pinto, Filippe E. F. Soares

**Affiliations:** 1 Instituto de Patologia Tropical e Saúde Pública, Universidade Federal de Goiás, Rua 235 s/n, Goiânia, Goiás 74690-900 Brazil; 2 Departamento de Química, Universidade Federal de Lavras, Lavras, Minas Gerais 37200-900 Brazil

**Keywords:** Biological control, Entomopathogenic fungi, Optimization, Enzymes, Riboflavin

## Abstract

Nutritional factors exert significant influence on the growth of entomopathogenic fungi, one of the main agents employed commercially in the biological control of arthropods. Thus, the objective of this work is to optimize the culture medium and solid fermentation time for production of proteases and conidia of *Metarhizium robertsii* ARSEF 2575 and to evaluate the interference of riboflavin and salts on virulence and resistance to abiotic stress factors. In the first step, nine groups were separated: negative control, positive control, and seven supplementation groups: ammonium nitrate, ammonium chloride, potassium nitrate, sodium nitrate, ammonium sulfate, ammonium phosphate, urea. Sodium nitrate showed significant difference in protease production at the time of 20 days of solid fermentation. Then, different concentrations of sodium nitrate and riboflavin as supplement were evaluated. Response surface methodology demonstrated that riboflavin and sodium nitrate influence proteolytic activity and conidia production, but without synergism. Supplementation of the medium with the optimal concentration of sodium nitrate and riboflavin did not interfere with the germination of conidia without exposure to abiotic stress, but did increase the thermotolerance of conidia. The presence of riboflavin and sodium nitrate at optimal concentrations in the culture medium did not alter fungal virulence with and without exposure to heat stress, varying according to the presence or absence of the supernatant during exposure, evidencing that resistance to heat exposure is multifactorial and dependent on intra- and extracellular factors. Moreover, the supplementation increased the larvicidal activity of the supernatant against *Aedes aegypti*.

**Supplementary Information:**

The online version contains supplementary material available at 10.1186/s13568-021-01326-1.

## Key points

- Riboflavin and NaNO_3_ increased *M. robertsii* protease and conidial production.
- The optimized medium increased the thermotolerance of *M. robertsii* conidia.
- Supplemented medium has positive influence on larvicidal action against *Ae. aegypti.*

## Introduction

The growing demand for sustainable methods of pest control has generated an increase in the consumption of organic products that act as biocontrollers, in particular microorganisms, especially entomopathogenic fungi. The *Metarhizium* genus, in particular, represents a significant portion of bioproducts marketed in several countries (Mascarin et al. 2019; Parra and Junior 2019).

Among the species of this genus, *Metarhizium robertsii* has shown promising results in several research fields (Paixão et al. 2021; Huarte-Bonnet et al. 2020; Lahey et al. 2020). Early in the infection process, the fungus secretes enzymes that catalyze the hydrolysis of the cuticle and allow hyphae to cross it and reach the hemocel (Guo et al. 2017).

Different compounds, such as inorganic salts and vitamins, can influence biomass formation and extracellular enzyme production in certain isolates (Shah et al. 2005). Studies related to the use of additives for *M. robertsii* are scarce, one of the most promising being riboflavin—a complex nitrogen source mainly related to cellular oxidative metabolism (Pereira-Junior et al. 2018). However, studies that determine in detail riboflavin as a potential supplement for *M. robertsii* culture media are lacking.

In this context, some arthropods have great importance for public health on the world stage and their control is of great importance. Dengue is the most prevalent arbovirus disease worldwide, transmitted by the *Aedes aegypti* mosquito. It is estimated to have over 390 million cases per year and over 20,000 deaths on the global stage, mainly in tropical countries (Wu et al. 2019; Anoopkumar et al. 2017).

Thus, the objective of this study was to optimize the culture medium for the production of proteases and conidia of *M. robertsii* ARSEF 2575 and to evaluate the interference of riboflavin and inorganic salts on the virulence of the fungus in *A. aegypti* larvae and the response to heat stress.

## Materials and methods

### Obtaining the fungal isolate

*Metarhizium robertsii* ARSEF 2575 is deposited at the United States Department of Agriculture – USDA-ARS Collection of Entomopathogenic Fungal Culture (Ithaca, NY, USA). The fungus was cultured in Petri dishes (80 × 15 mm) containing potato dextrose agar (BDA) medium for 15 days at 27 ± 1 °C, in a 12-h photoperiod (Alves et al. 1998a, b).

### Biphasic fermentation

The methodology used was an adaptation of that used by Dhar and Kaur (2010), Barra-Bucarei et al. (2016) and Lopes (2016). For the experiment, nine groups were formed: two control groups, one positive and one negative, and seven treated groups. A basal medium was produced with the following components: 100 mL of distilled water, 1% Tween 80® (0.1% v/v) and sucrose (20 g L^−1^). The negative control was composed of basal medium only, while the positive control was prepared with basal medium plus yeast extract (25 g L^−1^) as a nitrogen source.

The treated groups were prepared with the basal medium plus the addition of an inorganic salt. The salts evaluated were: ammonium nitrate (NH_4_NO_3_) (1.0 g L^−1^), ammonium chloride (NH_4_Cl) (1.34 g L^−1^), potassium nitrate (KNO_3_) (2.53 g L^−1^), sodium nitrate (NaNO_3_) (1.58 g L^−1^), ammonium sulfate [(NH_4_)_2_SO_4_] (3.0 g L^−1^), ammonium phosphate [(NH_4_)_3_PO_4_] (5.0 g L^−1^), urea (CH_4_N_2_O) (2.14 g L^−1^) (Sabbour 2002; Schamne 2010; Habte and Osorio 2012; Hamzah et al. 2012; Alves et al. 1998a, b).

The solutions of each group were prepared in Erlenmeyer flasks and autoclaved at 120 °C and 120 psi for 20 min. Then, each flask was inoculated with 1 cm^2^ of previously prepared BDA culture and kept under stirring at 150 rpm, 25 °C for 3 days.

Two polypropylene bags (25 × 35 × 0.6 cm) were used for each group, totaling eighteen bags. In each bag was placed 100 g of parboiled rice (40% moisture) (Lopes 2016) and 5 mL of prepared salt solution was added. All bags were autoclaved.

Subsequently, the rice was treated inside a laminar flow hood with 5 mL of the inoculum prepared in liquid fermentation, maintaining the composition of the groups. All rice bags were incubated at 27 °C in 12 h photoperiod. After the fifth day the rice was manually homogenized daily. After the incubation time had elapsed, the rice was washed with 500 mL of Tween 80® (0.1% v/v) solution and the suspension was used as sample for the subsequent tests.

### Conidia production and viability

Conidia production and viability was evaluated at two different incubation times, 10 or 20 days. To determine the amount of conidia produced, 20 µL aliquots of suspension were quantified in a Neubauer chamber. The quantification was done in duplicate.

To assess conidia viability, Petri dishes (35 × 10 mm) containing 10 mL of BDA plus 0.002% (w/v) benomyl and 0.05% (w/v) chloramphenicol (BDA + B + C) were prepared. The suspensions obtained from each bag were individually adjusted to the concentration of 1 × 10^6^ conidia mL^−1^, and 20 μL was inoculated in the center of the previously prepared plates (Milner et al. 1991; Braga et al. 2001).

The plates were incubated for 24 h at 27 ± 1 °C in a 12 h photoperiod. After this period, two drops of Amann's lactophenol and cotton blue were inoculated onto the sample in the culture medium. Then, it was observed under light microscopy at 400× magnitude and a minimum number of 300 conidia was evaluated per plate to calculate the percentage of germinated conidia (Braga et al. 2001).

### Protease assay

Proteolytic activity was measured by the caseinolytic method according to Feridi et al. (2011). The volumes of solutions used were: 50 μL of each sample (see in Biphasic fermentation) and 200 μL pH 8.0 McIlvaine buffer containing azocasein 1%. The reaction medium was incubated for 1 h at 37 °C and stopped by the addition of 600 μL of trichloroacetic acid (TCA) solution 10%. Samples were centrifuged at 10,000*g* for 10 min at 4 °C. Then, 600 μL of supernatant were transferred and neutralized with 700 μL of 1 N sodium hydroxide solution. The absorbance was determinate in an automatic microplate reader at 450 nm. A protease unit, named as “U”, was defined as the amount needed to increase absorbance at 450 nm by 0.01 after 60 min under the test conditions.

### Central composite design

The variables, enzyme activity and conidia production, were evaluated using response surface methodology to obtain the best combination of riboflavin and sodium nitrate levels.

To determine the optimal conditions for *M. robertsii* ARSEF 2575 production, central composite design (CCD) was applied. For the statistical design two variables were considered: riboflavin and inorganic salt, as shown in Table 1. Based on the results found in the previous experiments, sodium nitrate was used. The experimental design was used with four replicates at the central point, totaling twelve experiments for the investigation of the variables. To find the appropriate ranges, a variation of the high and low levels was made. Data analysis was performed using Design Expert 12 software.

**Table 1:** Highest and lowest levels (−1 a + 1) for the two variables: riboflavin (A) and sodium nitrate (B) used according to the experimental desing for Response Surface Methodology for optimization of protease and conidia production of *Metarhizium robertsii* ARSEF 2575

Variables	Variable Code	Lowest level (−1)	Higher level (+1)
Riboflavin % (g/g)	A	0.001	0.199
NaNO_3_% (g/g)	B	0.006	0.373

### Exposure to thermal stress

#### Biphasic fermentation in optimized medium

*Metarhizium robertsii* ARSEF 2575 was grown according to the methodology described in Biphasic fermentation, but the composition of medium was suitable for the concentration of riboflavin and sodium nitrate optimized as analyzed previously. Riboflavin and sodium nitrate were diluted in 5 mL of Tween 80® 0.1% (v/v) and added to parboiled rice before sterilization.

The suspension obtained from washing the rice was divided into three groups. The first group consisted of 150 mL of the crude extract from the washing. For the other groups, 150 mL of the crude extract was centrifuged and the conidia and other cells were separated from the supernatant of the suspension. The cells were suspended in Tween 80® 0.1% (v/v). Thus, three groups were formed: crude extract (complete suspension obtained from rice washing), conidia (only conidia suspended in Tween 80® 0.1% (v/v)) and supernatant (only metabolites and proteins suspended in the crude extract obtained from rice washing).

### Exposure to high temperature

The heat exposure methodology was adapted from Rangel et al. (2012). Fifty milliliters of the previously described suspensions (crude extract, cell suspension and supernatant) were added separately in 50 mL Falcon tubes and taken to a water bath at 45 ± 0.2 °C for 6 h. At each 60 min interval a 20 µL aliquot was inoculated in BDA + B + C medium to analyze the germination percentage. The exposure times were named: T0, T1, T2, T3, T4, T5 and T6. Three repetitions were evaluated.

For the analysis of proteolytic activity, one milliliter was collected from each group at exposure time T0 and T2. For the virulence test against *A. aegypti* larvae, fifty milliliters were used after 0 h and 2 h of exposure (T0 and T2).

### Conidial viability

The procedure previously described in Conidia production and viability was performed with an adjustment in the time and photoperiod of incubation of the plates: 48 h at 27 ± 1 ºC in the dark (Rangel et al. 2012). Three repetitions were evaluated.

### Virulence test against *Aedes aegypti* larvae

The mortality assay methodology was adapted from the method described by Falvo et al. (2018). For each treatment, it was used 10 s-stage larvae (L2) obtained from the parasitology department of Instituto de Patologia Tropical e Saúde Pública (IPTSP). They were placed in plastic cups (3.9 cm height × 4 cm diameter) containing 25 mL of suspensions previously prepared according to biphasic fermentation in optimized medium at 1 × 10^7^ conidia mL^−1^. A control group containing only Tween 80® 0.1% (v/v) was also performed. Larvae were fed every 2 days as described by Falvo et al. (2016), maintained at 27 ± 1 °C in 12 h photoperiod. Mortality was assessed for 10 days. Three replicates were evaluated. Dead larvae were incubated in water-agar (1% w/v) supplemented with chloramphenicol (0.05% w/v), thiabendazole (0.0004% w/v) and crystal violet (0.001% w/v) at 27 ± 1 °C for 25 days.

### Protein quantification

The concentration of soluble proteins in the supernatant group was quantified according to the Lowry method (Lowry et al. 1951) using bovine serum albumin (BSA) (Darmstadt, Germany, Merck®) as a standard. In a test tube, 0.2 mL of distilled water, 10 µL of the aliquot and 1 mL of alkaline solution (14.7 mL of 20 g Na_2_CO_3_/100 mL 1 N NaOH; 0.15 mL of potassium sodium tartrate; 0.15 mL of CuSO_4_) were placed. The tube was shaken and incubated for 15 min, and then 0.1 mL of Folin-Ciocalteou's reagent (diluted in a 0.1:1 v/v ratio in distilled water) was added. The samples were read in spectrophotometer at 660 nm absorbance. The standard curve was constructed using Bovine Serum Albumin (BSA) at concentrations of 5, 10, 15 and 20 µg/ µL according to the conditions described above. The quantification of the supernatant was performed with three repetitions.

### Statistical analysis

The results of proteolytic activity and conidia production, the regression model and the percentage of conidia germination and larval mortality were evaluated by analysis of variance (ANOVA) and Tukey's test considering *P* < 0.05 as significant. The software used was BioEstat 5.0 (Ayres et al. 2007).

## Results

### Conidia production on inorganic salt media

The analysis of the amount of conidia produced by the fungus in rice supplemented with different inorganic salts showed no significant difference (P > 0.05) between the groups treated among themselves or when compared to the control groups. This result was the same for the groups fermented for 10 days and for the groups fermented for 20 days. All groups showed viability above 97% and no statistical difference between them.

Also, there was no significant difference (P > 0.05) between the treatments with the same supplementation when fermented for different times, as shown in Table 2.

**Table 2:** Average conidial production of *Metarhizium robertsii* ARSEF 2575 in biphasic fermentation on basal medium supplemented with inorganic salts

Treatment	Conidial production (10^8^ conidia/mL) Fermentation 10 days	Conidial production (10^8^ conidia/mL) Fermentation 20 days
Ammonium nitrate	7.10 ± 2.7	7.40 ± 3.8
Ammonium chloride	6.35 ± 3.0	13.00 ± 12.0
Potassium nitrate	0.84 ± 0.2	2.61 ± 0.1
Sodium nitrate	3.59 ± 1.4	3.74 ± 3.2
Ammonium sulfate	4.85 ± 2.5	7.79 ± 6.6
Phosphorus nitrate	6.10 ± 4.9	9.83 ± 8.4
Urea	0.71 ± 0.4	1.92 ± 1.5
Negative control	1.44 ± 1.3	2.58 ± 1.3
Positive control	4.72 ± 1.1	5.05 ± 4.2

Conidia were collected after solid fermentation in parboiled rice for 10 or 20 days. Scale 1 × 10^8^ conidia/ mL

### Proteolytic activity in inorganic salt media

Enzymatic units (U) of proteases are shown in Table 3. The analysis of proteolytic activity after solid fermentation for 10 days showed higher activity only in the group supplemented with potassium nitrate compared to the group supplemented with ammonium chloride and urea (P < 0.05). The other groups showed no higher or lower proteolytic activity among themselves or compared to the control groups.

**Table 3:** Protease activity of *Metarhizium robertsii* ARSEF 2575 produced in medium supplemented with different inorganic salts and solid fermentation time

Treatment	Protease activity (U/mL) Fermentation—10 dias	Protease activity (U/mL) Fermentation—20 dias
Ammonium nitrate	10.7 ± 7.1^ac^	8.21 ± 4.4^ab^
Ammonium chloride	9.70 ± 4.0^a^	6.14 ± 0.8^a^
Potassium nitrate	19.7 ± 0.2^ac^	3.32 ± 0.3^a*^
Sodium nitrate	12.1 ± 0.1^bc^	23.1 ± 7.9^b^
Ammonium sulfate	11.4 ± 3.3^ac^	4.43 ± 1.8^a*^
Phosphorus nitrate	15.9 ± 1.4^ac^	9.16 ± 2.1^ab^
Urea	7.63 ± 0.1^a^	5.23 ± 1.6^a^
Negative control	12.9 ± 0.8^ac^	3.72 ± 1.2^a^
Positive control	14.8 ± 5.1^ac^	1.92 ± 1.3^a*^

Protease essay was evaluated after biphasic fermentation with solid fermentation in parboiled rice for 10 or 20 days

Lowercase letters refer to comparison between different components in culture medium, where averages followed by equal letters in the same column do not differ significantly from each other (P > 0.05)

*Refer to comparison between different components in the culture medium, where averages in the same column differ significantly in relation to the group supplemented with sodium nitrate (P > 0.01)

The analysis of proteolytic activity after fermentation for 20 days showed that proteolytic activity in group supplemented with sodium nitrate was higher than proteolytic activity of ammonium chloride and urea (P < 0.05), potassium nitrate and ammonium sulfate (P < 0.01) groups. In addition, sodium nitrate was higher compared to the negative control (P < *0*.05) and positive control (P < 0.01) groups. Thus, sodium nitrate was the inorganic salt chosen for comparative analysis with the effects of riboflavin.

## Response surface methodology

The results of proteolytic activity and conidia production from the 12 trials performed to determine the optimal levels of the two variables are shown in Table 4.

**Table 4:** Experimental design with 12 trials used to perform the response surface methodology

Races	Riboflavin (%)	NaNO_3_ (%)	Proteolytic activity (U/mL)	Conidia/mL (10 ^9^)
1	0.001	0.006	14.050	10.350
2	0.199	0.006	4.150	7.300
3	0.001	0.310	10.633	16.30
4	0.199	0.310	5.400	4.30
5	0	0.158	16.750	17.90
6	0.240	0.158	12.450	13.350
7	0.1	0	4.700	9.150
8	0.1	0.373	2.700	6.450
9	0.1	0.158	9.4	10.850
10	0.1	0.158	14.683	12.950
11	0.1	0.158	12.95	15.800
12	0.1	0.158	14.933	15.050

Two variables were used (riboflavin and sodium nitrate) each with 5 levels and their respective values of proteolytic activity and conidia production of *Metarhizium robertsii* ARSEF 2575

Table 5 shows the results of the F-test used to test the statistical significance of the regression model and the analysis of variance (ANOVA) used to obtain the quadratic response surface model.

**Table 5:** Analysis of variance for the response equation developed in the optimization of proteases production by *Metarhizium robertsii* ARSEF 2575

Source	Sum of squares	Degree of freedom	Mean Square	F value	P-value
Model	229.3	5	45.86	9.010	0.001
A-Riboflavin	56.26	1	56.26	11.06	0.016
B-Sodium nitrate	3.120	1	3.120	0.613	0.463
AB	5.440	1	5.440	1.070	0.341
A^2^	2.760	1	2.760	0.542	0.489
B^2^	147.0	1	147.0	28.90	0.002
Residual	30.53	6	5.090		
Lack of Fit	11.00	3	3.670	0.563	0.676
Pure Error	19.53	3	6.510		
Cor Total	259.8	11			

For the proteolytic activity model, the F value of 9.01 indicates that the model is significant. P values less than 0.05 indicate that the model terms are significant. In this case, A, B^2^ are significant model terms. The R^2^ of the model was 0.88 demonstrating the reliability of the quadratic model.

The final answer that provides the proteolytic activity of ARSEF 2575, can be obtained by the equation:

$${\text{Activity}}{:}{\text{ }}\left[ { + {\text{13}}.0{\text{453}}0 - {\text{52}}.{\text{411}}0{\text{1 Riboflavin }} + {\text{53}}.~{\text{5481}}0{\text{ Sodium nitrate }} + {\text{ 77}}.{\text{4278}}0{\text{ Riboflavin }}*{\text{ Sodium nitrate }} + {\text{6}}.{\text{95745 Riboflavin}}^{2} ~ - {\text{ 2}}0{\text{6}}.{\text{94}}0{\text{78 Sodium nitrate}}^{2} } \right].$$

**Fig. 1:**
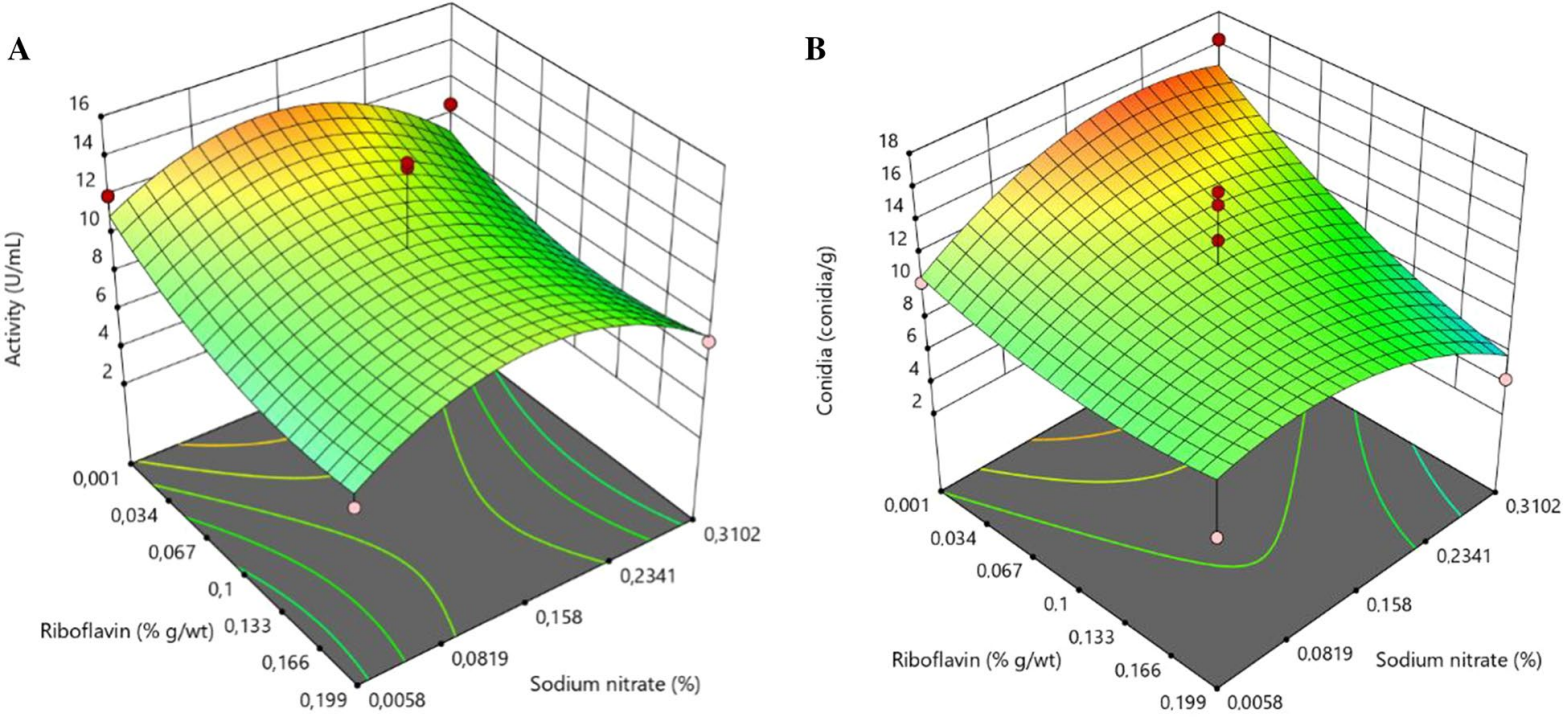
Response surface plots of *Metarhizium robertsii* ARSEF 2575 produced in parboiled rice supplemented with riboflavin and sodium nitrate. **A** Response surface plot of *Metarhizium robertsii* ARSEF 2575 for proteolytic activity. **B** Response surface plot for conidial production of *Metarhizium robertsii* ARSEF 2575. The unit measurement used was gram by weight (g/wt)

Figure 1A shows the three-dimensional response surface model according to the final model of the proteolytic activity of *M. robertsii* ARSEF 2575 produced in parboiled rice supplemented with riboflavin and sodium nitrate.

The quadratic model analysis revealed that both variables interfere in the proteolytic activity of the fungus, however, they do not act synergistically. The value of riboflavin concentration that resulted in higher proteolytic activity was 0.001% (g/p) while the ideal value of sodium nitrate concentration was approximately 0.158% (g/p). The increase in the concentration of these components in the medium did not mean an increase in the enzymatic activity, with a tendency to decrease it when in excess in the medium.

For the conidia production model, the F-value was 5.01 indicating that the model is significant. P-value lower than 0.050 indicates that the model terms are significant. Thus, also in this analysis, A and B^2^ are significant model terms. The R^2^ of the model was 0.80, which demonstrates that the quadratic model is reliable. The final response, which gives the conidia production of *M. robertsii* ARSEF 2575, can be obtained by the equation:

$${\text{Conidia}}{:}{\text{ }}\left[ {{\text{1}}0.{\text{85627 }} - {\text{ 12}}.{\text{72}}00{\text{3 Riboflavin }} + {\text{ 61}}.{\text{45484 Sodium nitrate }} - {\text{148}}.{\text{49547 Riboflavin }}*{\text{ Sodium nitrate }} + {\text{45}}.{\text{27599 Riboflavin}}^{2} {\text{ }} - {\text{ 149}}.{\text{742}}0{\text{2 Sodium nitrate}}^{2} } \right].$$

Again, attention is drawn to the fact that an equation in terms of real factors can be used to make predictions about the response for given levels of each factor, but the levels must be specified in the original units of each factor.

Figure 1B shows the three-dimensional response surface model according to conidia production of *M. robertsii* ARSEF 2575 produced on parboiled rice supplemented with riboflavin and sodium nitrate.

The quadratic model analysis revealed that both variables interfere in the fungus conidia production, however, they do not act synergistically. The ideal value of riboflavin concentration was 0.001% (g/p) while the ideal value of sodium nitrate concentration was between 0.158 and 0.2341% (g/p). Excessive increase in the concentration of these components in the medium showed a tendency to decrease conidia production. The optimal levels considering conidia production and proteolytic activity were 0.001% (g/p) for riboflavin and 0.162% (g/p) for sodium nitrate.

### Germination after heat stress

**Fig. 2:**
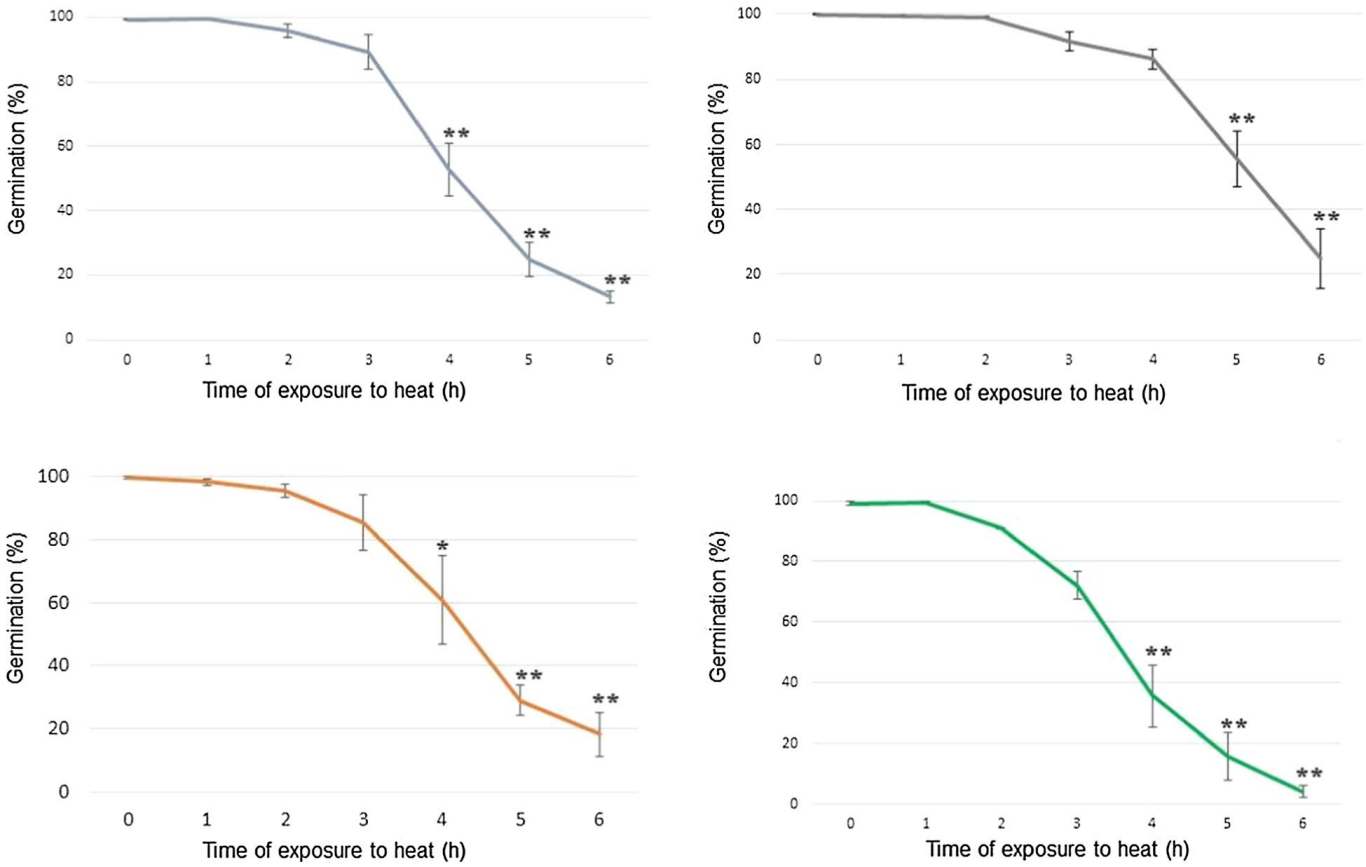
Relative viability (%) of *Metarhizium robertsii* ARSEF 2575 produced in parboiled rice supplemented or unsupplemented with riboflavin and sodium nitrate after exposure to heat stress. Rice was washed with Tween 80® (0.01% v/v) after twenty days of solid fermentation. The groups evaluated were: A: Crude extract produced in supplemented medium; B: Conidia produced in supplemented medium; C: Crude extract produced in unsupplemented medium; D: Conidia produced in unsupplemented medium. Samples of each group were exposed to 45 ± 0.2 °C and at each 1 h of exposure an aliquot was inoculated in culture medium for germination analysis. All exposure times were compared to the 0 h of heat exposure. *P < 0.05 and **P < 0.01 according to Tukey’s Test

**Fig. 3:**
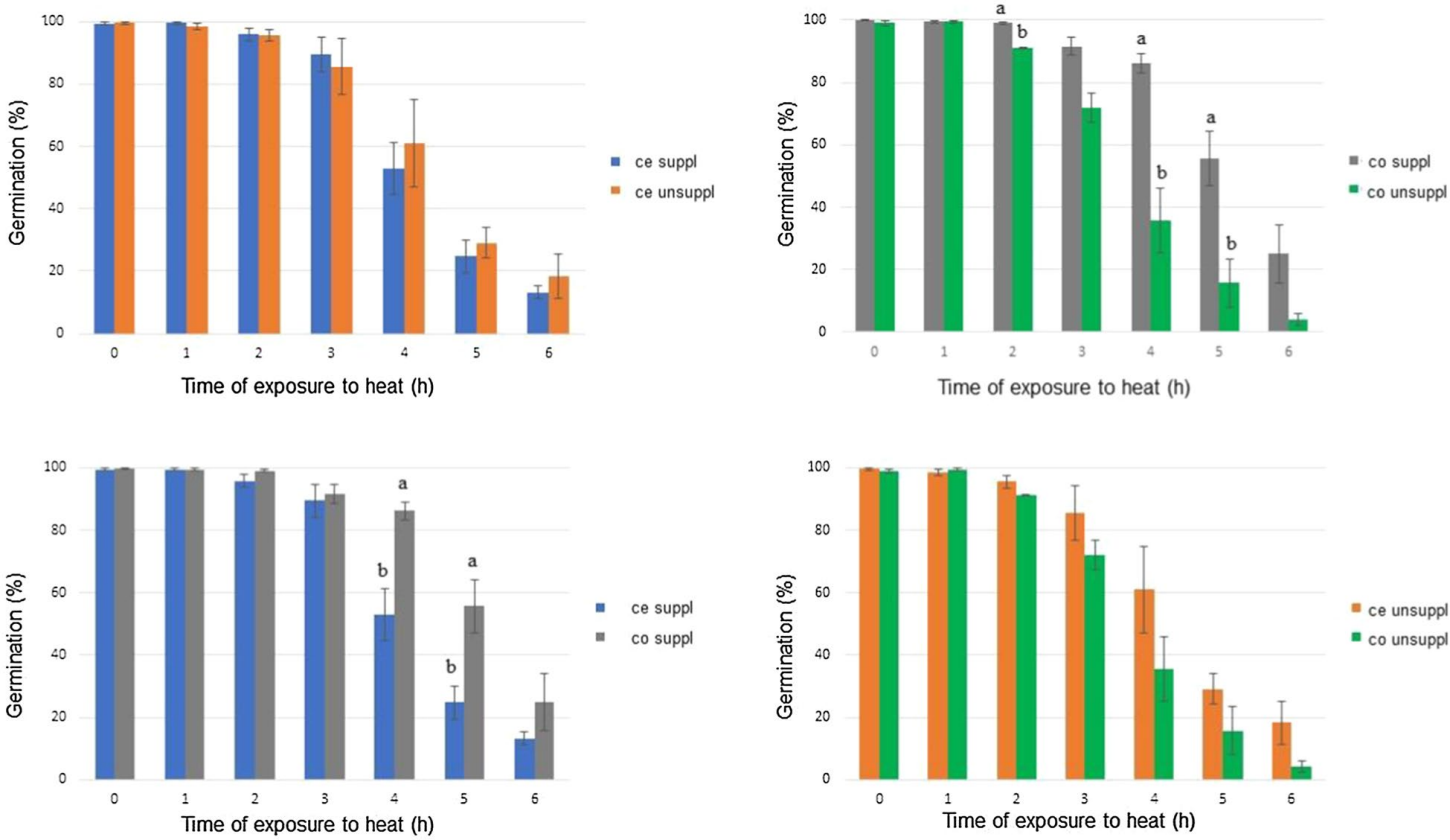
Comparison between relative viability (%) of *Metarhizium robertsii* ARSEF 2575 produced in parboiled rice supplemented and unsupplemented with riboflavin and sodium nitrate after exposure to heat stress. Rice was washed with Tween 80® (0.01% v/v) after twenty days of solid fermentation. One part of the crude extract was kept and the other part was centrifugates, filtered and conidia were resuspended in Tween 80® (0.01% v/v). Samples of each group were exposed to 45 ± 0.2 °C and at each 1 h of exposure an aliquot was inoculated in culture medium for germination analysis. Different treatments were compared at the same exposure times: A: Crude extract produced in supplemented and unsupplemented medium; B: Conidia produced in supplemented and unsupplemented medium; C: Crude extract and conidia produced in supplemented medium; D: Crude extract and conidia produced in unsupplemented medium. Samples of each group were exposed to 45 ± 0.2 °C and at each 1 h of exposure an aliquot was inoculated in culture medium for germination analysis. Different letters indicates *P < 0.05 according to Tukey’s Test

By analyzing the percentage of germination, it was observed that for all groups (crude extract and conidia produced in supplemented and unsupplemented medium) there was a reduction in the germination rate after 4, 5, and 6 h of exposure to 45 °C, except for the group of conidia produced in supplemented medium, which showed a significant reduction only after 5 and 6 h of exposure to heat, as shown in Fig. 2. The comparative analysis of the percentage of germination between the same treatments (crude extract or conidia) produced in supplemented and unsupplemented medium is shown in Fig. 3.

Comparison between crude extract produced in supplemented medium and crude extract produced in unsupplemented medium showed no significant difference (P > 0.05) between them at all exposure times. On the other hand, inoculum containing only conidia produced on supplemented medium showed higher (P < 0.05) germination percentage than inoculum containing only conidia produced on unsupplemented medium at the 2 h, 4 h, and 5 h exposure times.

In addition, a comparison was made between crude extract treatment and only conidia produced in the same type of medium. Conidia group showed a higher percentage of germination at 4 h and 5 h exposure time compared to crude extract group, both produced in medium supplemented with riboflavin and sodium nitrate. Between crude extract and conidia-only treatment groups produced on unsupplemented medium, there was no significant difference at any of the exposure times evaluated.

### Virulence test on *Aedes aegypti* larvae

Statistical analysis of mortality of *A. aegypti* larvae was performed on three days of evaluation. The three prepared suspensions (crude extract, conidia or supernatant) were considered as individual treatments. Each treatment was compared with the other two extracted from culture under similar culture medium conditions (supplemented with sodium nitrate and riboflavin or not) and stress conditions (exposed or not exposed to heat). Each treatment was also compared with the same treatment taken from culture under different culture condition (comparison between supplemented and unsupplemented medium).

Both treatments with supernatant, produced in supplemented and unsupplemented medium, not exposed to heat, showed a lower percentage of cumulative mortality than treatments with crude extract and conidia only, and showed no difference between them. This result was repeated on days 5 and 10 after treatment.

On day 3, among the treatments exposed to heat in unsupplemented medium, the treatment with supernatant showed a lower percentage of cumulative mortality compared to the treatment with conidia.

On days 5 and 10, among the heat-exposed treatments, the supernatant group produced in unsupplemented medium showed lower mortality compared to the other two heat-exposed treatments produced in unsupplemented medium and also with the heat-exposed supernatant treatment produced in supplemented medium, as shown in Table 6.

**Table 6:** Cumulative mortality of *Aedes aegypti* larvae after treatment with *Metarhizium robertsii* ARSEF 2575

Treat	Stress	SUPPL	UNSUPPL	Treat	Stress	SUPPL	UNSUPPL	Treat	Stress	SUPPL	UNSUPPL
		Mortality (%)				Mortality (%)				Mortality (%)	
		3 days				5 days				10 days	
Crude extract	None	66.7 ± 8.8^Aa^	80.0 ± 5.8^Aa^	Crude extract	None	90.0 ± 5.8^Aa^	96.7 ± 3.3^Aa^	Crude extract	None	100 ± 0^Aa^	100.0 ± 0^Aa^
Conidia	None	60.0 ± 5.8^Aa^	66.7 ± 12.0^Aa^	Conidia	None	80.0 ± 100 ^Aa^	90.0 ± 5.8^Aa^	Conidia	None	100 ± 0^Aa^	96.7 ± 3.3^Aa^
Supernatant	None	15.0 ± 5.0^Ab^	15.0 ± 5.0^Ab^	Supernatant	None	15.0 ± 5.0^Ab^	20.0 ± 0^Ab^	Supernatant	None	30.0 ± 10.0^Ab^	35.0 ± 5.0^Ab^
Crude extract	Heat	60.0 ± 5.8^Aa^	50.0 ± 5.8^Aab^	Crude extract	Heat	76.0 ± 3.3^Aa^	83.3 ± 120 ^Aa^	Crude extract	Heat	93.3 ± 3.3^Aa^	96.7 ± 3.3^Aa^
Conidia	Heat	53.3 ± 3.33^Aa^	66.7 ± 6.7^Aa^	Conidia	Heat	83.3 ± 6.7^Aa^	90.0 ± 5.8^Aa^	Conidia	Heat	100 ± 0^Aa^	96.7 ± 3.3^Aa^
Supernatant	Heat	70.0 ± 20.0^Aa^	15.0 ± 15.0^Bb^	Supernatant	Heat	70.0 ± 20.0^Aa^	25.0 ± 15^Bb^	Supernatant	Heat	90.0 ± 0^Aa^	45.0 ± 5.0^Bb^
Control		0 ± 0		Control		10 ± 3.3		Control		10 ± 3.3	

After twenty days of solid fermentation, rice was washed with Tween 80® (0.1% v/v). One part of crude extract was kept and the other part was centrifuged and filtered. Conidia were resuspended in Tween 80® (0.1% v/v) and supernatant stored. The three treatments (crude extract. conidia and supernatant) were separated into two subgroups: one kept at 27 ± 1 °C and the other exposed to 45 ± 0.2 °C for 2 h. Larvae were treated with the three types of treatments after biphasic fermentation in media supplemented (SUPPL) with riboflavin and sodium nitrate or unsupplemented media (UNSUPPL). The same treatments produced in different culture media or different treatments submitted to the same stress conditions were compared. Larvae were incubated at 25 ± 1 °C. Relative humidity greater than 75% and 12 h photoperiod for 10 days

Equal capital letters in the same row indicate no significant difference and equal lower-case letters in the same column indicate no significant difference according to Tukey's test (P < 0.05)

For these three days evaluated, a comparison was also made between the same treatments, but submitted to different stress conditions. In the three compared times, treatments with supernatant exposed and not exposed to heat were the only ones that presented significant difference, and the supernatant exposed to heat presented higher mortality percentage than supernatant not exposed.

The only treatment with supernatant that showed significantly higher mortality percentage than the control group on the three days evaluated was the supernatant extracted from supplemented medium and exposed to heat (P > 0.01 by Tukey's test), as shown in Table 7.

**Table 7:** Comparison between cumulative mortality of *Aedes aegypti* larvae after treatment with *Metarhizium robertsii* ARSEF 2575

Treat	Stress	SUPPL	UNSUPPL	Treat	Stress	SUPPL	UNSUPPL	Treat	Stress	SUPPL	UNSUPPL
		Mortality (%)				Mortality (%)				Mortality (%)	
		3 Days				5 Days				10 Days	
Crude extract	None	66.7 ± 8.8^A^	80.0 ± 5.8^A^	Crude extract	None	90.0 ± 5.8^A^	96.7 ± 3.3^A^	Crude extract	None	100 ± 0^A^	100.0 ± 0^A^
Crude extract	Heat	60.0 ± 5.8^A^	50.0 ± 5.8^A^	Crude extract	Heat	76.0 ± 3.3^A^	83.3 ± 12.0^A^	Crude extract	Heat	93.3 ± 3.3^A^	96.7 ± 3.3^A^
Conidia	None	60.0 ± 5.8^A^	66.7 ± 12.0^A^	Conidia	None	80.0 ± 10.0^A^	90.0 ± 5.8^A^	Conidia	None	100 ± 0^A^	96.7 ± 3.3^A^
Conidia	Heat	53.3 ± 3.33^A^	66.7 ± 6.7^A^	Conidia	Heat	83.3 ± 6.7^A^	90.0 ± 5.8^A^	Conidia	Heat	100 ± 0^A^	96.7 ± 3.3^A^
Supernatant	None	15.0 ± 5.0^A^	15.0 ± 5.0^A^	Supernatant	None	15.0 ± 5.0^A^	20.0 ± 0^A^	Supernatant	None	30.0 ± 10.0^A^	35.0 ± 5.0^A^
Supernatant	Heat	70.0 ± 20.0^B^	15.0 ± 15.0^A^	Supernatant	Heat	70.0 ± 20.0^B^	25.0 ± 15^A^	Supernatant	Heat	90.0 ± 0^B^	45.0 ± 5.0^A^
Control		0 ± 0		Control		10 ± 3.3		Control		10 ± 3.3	

After twenty days of solid fermentation, rice was washed with Tween 80® (0.1% v/v). One part of crude extract was kept and the other part was centrifuged and filtered. Conidia were resuspended in Tween 80® (0.1% v/v) and supernatant stored. The three treatments (crude extract. conidia and supernatant) were separated into two subgroups: one kept at 27 ± 1 °C and the other exposed to 45 ± 0.2 °C for 2 h. Larvae were treated with the three types of treatments after biphasic fermentation in media supplemented (SUPPL) with riboflavin and sodium nitrate or unsupplemented media (UNSUPPL). The same treatments produced on different culture media or subjected to the same stress conditions were compared. Larvae were incubated at 25 ± 1 °C. Relative humidity greater than 75% and 12 h photoperiod for 10 days

Equal capital letters in the same column indicate no significant difference according to Tukey's Test (P < 0.05)

Also, a photographic record of the cadaver of the groups treated with crude extract and conidia was taken to confirm the fungal infection 25 days after treatment as shown in Additional file 1: Fig. S1 (treated with unheated conidia) and Additional file 1: Fig. S2 (treated with heat conidia).

### Protein quantification

A higher amount of soluble protein was measured in the supernatant extracted from fungus grown on supplemented medium than supernatant extracted from fungus grown on unsupplemented medium (P < 0.05).

## Discussion

The results of the tests of supplementing the culture medium with different inorganic nitrogen sources showed that the tested salts are promising, since almost all of them (except potassium nitrate and urea at the 10-day solid fermentation time) reached production in the range of 10^8^ conidia/mL. However, these components alone were not sufficient to reach an optimal production level, which is at least 10^9^ conidia/mL range, as described by Loera-Corral et al. (2016).

Thus, riboflavin supplementation was extremely important for optimization of fungal culture medium. The joint supplementation of riboflavin with sodium nitrate has shown to be efficient in inducing conidia production in the range of 10^10^ conidia/mL, an amount considered optimal compared to other studies of production optimization of fungi of the genus *Metarhizium* (Loera-Corral et al. 2016; Barra-Bucarei et al. 2016; Prakash et al. 2008).

Conidia production is a commonly performed analysis in media optimization work for entomopathogenic fungi production using response surface methodology (Loera-Corral et al. 2016; Barra-Bucarei et al. 2016; Prakash et al. 2008; Bich et al. 2018). This methodology is also used for optimization of media in solid fermentation for the production of chitinases and cellulases (Aita et al. 2019). The results of this study show that this methodology is efficient in the search for the optimization of the culture medium not only in terms of the production of biomass, conidia and chitinases, but also regarding the production of proteases.

By means of biphasic fermentation method, significant total protease activity was obtained. The average proteolytic activity was significant as described by Dhar and Kaur (2010), who produced different isolates of *Metarhizium anisopliae* in liquid fermentation system containing 1% casein and yeast extract as substrate. This suggests that biphasic fermentation generates significant results regarding the production of total proteases (among which also specific proteases for cuticle degradation), and further studies using this technique are needed, since it is the closest to the one used by entomopathogenic fungi producers in Brazil (Jaronski 2014; Santos 2017).

Thermotolerance of *M. robertsii* ARSEF 2575 has not been described in the literature so far when the fungus is grown on parboiled rice, only on BDAY medium. The results of this study showed no significant reduction in the percentage of conidia germination until the time of 4 h of exposure at 45 °C. The result evaluated by Rangel et al. (2005), however, showed a decrease of this percentage for the same fungal species after 2 h of exposure at 45 °C. While the germination of the fungus cultivated in rice, supplemented or not, had a percentage higher than 80% in the 2 h exposure time and higher than 40% in the 4 h exposure time, the same test performed by Rangel et al. (2005) with fungus cultivated in BDAY showed a percentage lower than 60% in the 2 h exposure time and lower than 20% in the 4 h exposure time. This comparison suggests that this species, when grown on parboiled rice, presents higher thermotolerance.

At the 4 h exposure time, there was a significant difference in the percentage of relative germination between the conidia group (higher than 80%) and the crude extract (close to 50%) exposed to heat when obtained from rice supplemented with sodium nitrate and riboflavin. Both treatments had a higher value than that obtained by Paixão et al. (2017), who, despite exposing the same species to the same conditions described by Rangel et al. (2005), obtained a germination percentage close to 40% at the 4 h exposure time. The results of germination at 4 h for crude extract and conidia produced in unsupplemented rice did not differ significantly from each other (60% and 35% respectively), being these, therefore, results closer to the described by Paixão et al. (2017) in the same conditions.

Rangel et al. (2005) and Paixão et al. (2017) exposed conidia suspended in Tween 80® (without supernatant interference) to heat stress, thus, the differences with the data of the present work suggest that rice supplementation contributes not only to thermotolerance but also the composition of the supernatant has an influence on the thermotolerance of the fungus in longer times of exposure. Although both treatments showed a higher percentage of germination in supplemented medium compared to literature data, the treatment where only the conidia were exposed to heat showed a higher percentage. This suggests that the removal of some component of the supernatant allowed a higher percentage of relative germination of conidia at 4 h and 5 h.

Thus, the evaluation of in vivo virulence assay helped to clarify if the presence of some component in the supernatant induced by rice supplementation contributed to the virulence of the fungus. In the tests performed with *A. aegypti* larvae, the group treated with supernatant alone stood out at all compared times, showing lower lethality. Mortality of the supernatant obtained from both media (supplemented and unsupplemented) not exposed to heat did not differ from the untreated control. This suggests that larval death occurred as a result of fungal infection and not only by the action of enzymes or secondary metabolites contained in the supernatant, as described by Butt et al. (2013) and Vivekanandhan et al. (2020) for the action of *M. anisopliae* grown in sabouraud, dextrose, agar (SDA) medium on *A. aegypti* larvae.

The treatment with culture supernatant in supplemented medium and exposed to heat, however, showed significant mortality similar to the groups treated with crude extract and conidia produced in supplemented medium, and higher than the supernatant not exposed to heat on the three days evaluated. This treatment was also superior to the exposed supernatant produced in non-supplemented medium. This result indicates that supplementation of the culture medium with riboflavin and sodium nitrate had an influence on the response of the fungus during heat exposure-releasing compounds in the extracellular medium (supernatant) that could have insecticidal activity. Due to the absence of fungal cells, this result is corroborated by the description of lethality caused by enzymes and metabolites in the suspension (Butt et al. 2013; Vivekanandhan et al. 2020). Thus, these results are an indication that supplementation of the medium may lead to larval death by the association of fungal infection with enzymes and metabolites produced after heat stress for *A. aegypti* larvae.

Riboflavin and sodium nitrate are already used for media supplementation of entomopathogenic fungi (Iwanicki et al. 2020), but there are no reports of these components alone and their influence on the thermotolerance of the fungus. Pereira-Junior et al. (2018) demonstrated that supplementation of BDA medium with riboflavin increased expression of photolyase, laccase, and polyketide synthase genes in *M. robertsii* ARSEF 2575. Thus, just as the presence of riboflavin in BDA induced the expression of components that increase tolerance to UV radiation, the presence of riboflavin in rice may induced fungal proteins production. However further studies are needed to elucidate which proteins these are and whether they were induced by the presence of riboflavin or by the composition of rice.

Exposure of fungi *Metarhizium* sp. to high temperatures induces expression of heat shock proteins, many of which promote the folding of proteins responsible for repairing damage in the cell (Lovett and St. Leger 2015). Riboflavin, in turn, acts as a cofactor of FMN and FAD molecules, molecules that are essential for redox homeostasis, protein folding, DNA repair, β-oxidation of fatty acids, and amino acid oxidation (Liu et al. 2020). Thus, the presence of riboflavin in the culture medium could favor fungal thermotolerance, as well as induced tolerance to UV radiation (Pereira-Junior et al. 2018), by the increase of proteins responsible for cell repair. In addition to intracellular proteins, possible extracellular components contributing to heat tolerance, since riboflavin increased the expression of genes involved in conidia pigmentation and therefore radiation tolerance (Pereira-Junior et al. 2018; Lovett and St. Leger 2015). With all this in mind, this study demonstrated that the presence of riboflavin and sodium nitrate increased *M. robertsii* thermotolerance.

## Supplementary Information

**Additional file 1.** **Figure S1**. Aedes aegypti larvae treated with Metarhizium robertsii ARSEF 2575 not exposed to heat stress. Fungi was cultivated in parboiled ricesupplemented or unsupplemented with riboflavin and sodium nitrate. Rice was washed with Tween 80® (0,01% v/v) twenty days after solid fermentation: crudeextract and conidia resuspended in Tween 80® (0,01% v/v) were used to larval treatment. Photographs taken 25 days after treatment.**Figure S2**. Aedes aegypti larvae treated with Metarhizium robertsii ARSEF 2575 exposed to heat stress. Fungi was cultivated in parboiled rice supplemented orunsupplemented with riboflavin and sodium nitrate. Rice was washed with Tween 80® (0,01% v/v) twenty days after solid fermentation: crude extract and conidia resuspended in Tween 80® (0,01% v/v) were exposed to 45 ± 0,2°C in a water bath for 2 hours and then used to larval treatment. Photographs taken 25 days after treatment.

## Data Availability

*M. robertsii* ARSEF 2575 is deposited at the United States Department of Agriculture – USDA-ARS Collection of Entomopathogenic Fungal Culture (Ithaca, NY, USA).
