# Supplementary material for: Metarhizium robertsii protease and conidia production, response to heat stress and virulence against Aedes aegypti larvae
Source: AMB Express. 2021 Dec 13;11:166. doi: 10.1186/s13568-021-01326-1 (PMC8669065; doi:10.1186/s13568-021-01326-1)
Supplement: Supplementary file 1 — Additional file 1. Figure S1. Aedes aegypti larvae treated with Metarhizium robertsii ARSEF 2575 not exposed to heat stress. Fungi was cultivated in parboiled ricesupplemented or unsupplemented with riboflavin and sodium nitrate. Rice was washed with Tween 80® (0,01% v/v) twenty days after solid fermentation: crudeextract and conidia resuspended in Tween 80® (0,01% v/v) were used to larval treatment. Photographs taken 25 days after treatment.Figure S2. Aedes aegypti larvae treated with Metarhizium robertsii ARSEF 2575 exposed to heat stress. Fungi was cultivated in parboiled rice supplemented orunsupplemented with riboflavin and sodium nitrate. Rice was washed with Tween 80® (0,01% v/v) twenty days after solid fermentation: crude extract and conidia resuspended in Tween 80® (0,01% v/v) were exposed to 45 ± 0,2°C in a water bath for 2 hours and then used to larval treatment. Photographs taken 25 days after treatment. [file 13568_2021_1326_MOESM1_ESM.pdf]

Applied Microbiology and Biotechnology

Metarhizium robertsii protease and conidia production, response to heat stress and virulence against *Aedes aegypti* larvae

Juliana M. Ferreira[1]; Salorrane M. N. Pinto[1]; Filippe E.F. Soares[2*]

[1] Instituto de Patologia Tropical e Saúde Pública, Universidade Federal de Goiás, Rua 235 s/n, Goiânia, Goiás, Brazil. 74690-900.

[2] Departamento de Química, Universidade Federal de Lavras, Minas Gerais, Brazil, 37200-900.

*To whom correspondence should be addressed, at:

e-mail: filippe.soares@ufla.br

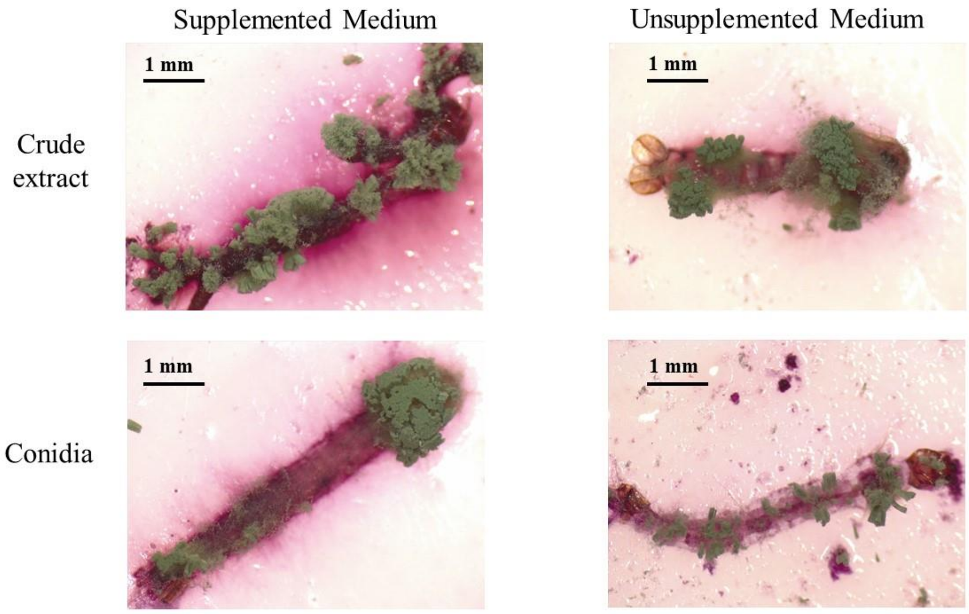

Figure S1. *Aedes aegypti* larvae treated with *Metarhizium robertsii* ARSEF 2575 not exposed to heat stress. Fungi was cultivated in parboiled rice supplemented or unsupplemented with riboflavin and sodium nitrate. Rice was washed with Tween 80® (0,01% v/v) twenty days after solid fermentation: crude extract and conidia resuspended in Tween 80® (0,01% v/v) were used to larval treatment. Photographs taken 25 days after treatment.

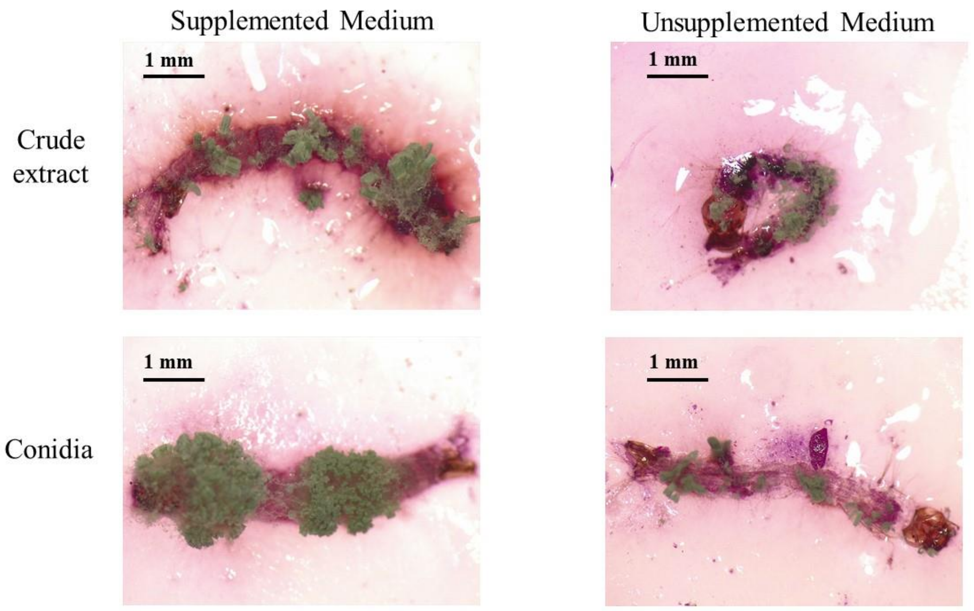

Figure S2. *Aedes aegypti* larvae treated with *Metarhizium robertsii* ARSEF 2575 exposed to heat stress. Fungi was cultivated in parboiled rice supplemented or unsupplemented with riboflavin and sodium nitrate. Rice was washed with Tween 80® (0,01% v/v) twenty days after solid fermentation: crude extract and conidia resuspended in Tween 80® (0,01% v/v) were exposed to 45 ± 0,2°C in a water bath for 2 hours and then used to larval treatment. Photographs taken 25 days after treatment.
